# Sport Courage, Worry and Fear in Relation to Success of Alpine Ski Learning

**DOI:** 10.3390/sports6030096

**Published:** 2018-09-14

**Authors:** Vjekoslav Cigrovski, Ivan Radman, Erkut Konter, Mateja Očić, Lana Ružić

**Affiliations:** 1 Faculty of Kinesiology Horvacanski zavoj, University of Zagreb, Zagreb 10000, Croatia; ivan.radman@kif.hr (I.R.); mateja.ocic@kif.hr (M.O.); lana.ruzic@kif.hr (L.R.); 2 Sport Science and Technology High School, Dokuz Eylül University, Izmir 35220, Turkey; erkut.konter@gmail.com

**Keywords:** sport courage, self-efficiency, worry, fear, skiing performance

## Abstract

(1) Background: Individuals’ psychological traits can influence not just success in sport but also the ability to learn new motor skills. We investigated whether sport courage, worry and fear differ between alpine ski-naive and basic level skiers and how they affect ski learning. (2): A total of 337 students (249 ski-naive and 88 basic level) participated in research consisting of a four-part questionnaire and structured skiing program. (3) Results: For beginners, lower fear (*r =* −0.30, *p <* 0.01) and higher Self-efficiency (*r =* 0.28, *p <* 0.05) and mastery (*r =* 0.20, *p <* 0.01) were associated with better performance; reducing fear and increasing self-efficiency and worry increased performance. Experienced skiers were better in determination, mastery, and self-efficiency (all *p <* 0.05). In case of lower score in worry (*r =* −0.28, *p <* 0.01) and higher in self-efficiency (*r =* 0.22, *p <* 0.05) performance was better. Males scored higher in sport courage scale-31 (all *p <* 0.05). In particular, self-efficiency was associated with better (*r =* 0.39, *p <* 0.01), and higher fear with poorer performance (*r =* −0.33, *p <* 0.01). Moreover, self-efficiency was a predictor of ski success (*p <* 0.001). On the other hand, females like ski beginners scored higher in fear (*p <* 0.001). In females, determination, mastery and self-efficiency had a positive correlation with skiing (*r =* 0.21, *p <* 0.05, *r =* 0.28, *p <* 0.01, and *r =* 0.33, *p <* 0.01, respectively), while association between Fear and skiing (*r =* −0.46, *p <* 0.01) was negative, and fear (*p <* 0.001) was inversely related to success. (4): Conclusions: Psychological factors and gender differences need to be considered during learning phases of alpine skiing. There is a positive association between self-efficiency and performance of male ski beginners, and negative association between fear and achieved results in basic alpine ski school in case of female ski beginners.

## 1. Introduction

Courage, self-efficiency, worry, anxiety, and fear have clinical importance and can be associated with different medical conditions, alter every-day life, but can also greatly influence individual’s success in sport [1]. The latter has received much attention in sport literature, primarily related to investigation of factors influencing success on a professional level. The literature suggests fear, anxiety and worry usually have detrimental influence on performance, while self-efficiency and courage, historically perceived as virtue, are nowadays treated as skills and instruments that can have a dramatically positive effect on competitive sport [2,3,4,5,6]. Moreover, the term sport courage relates to the ability of a person to be competent, determined, assertive, and make sacrifice on voluntary basis and in challenging circumstances to achieve a sport success/result [7]. 

Sport courage is a dynamic process, influenced by numerous factors such as situations (e.g., danger, fear, risk), type of sport, personal traits, previous experience, and type of the task the athlete is confronted with [8]. Currently, sports psychology focuses on different psychological variables influencing athletes’ performance, with the intention of maximizing efficiency [6]. Psychological outcomes have social foundations that are sometimes gender-related, which means that gender can have an important role in linking psychological factors and performance. According to this view, boys are taught to be more competitive and encouraged to take risks; also they are more self-confident and thus conditioned to be efficacious [9]. Teachers and coaches may contribute to mentioned factors by having different expectations from females and males, and by providing different practice opportunities, which finally can lead to men being more confident in their sport abilities and prone to participate in activities promoting development of motor skills associated with sports. Moreover, boys and men can be more encouraged to take part in adventurous and extreme sports [2,10,11]. 

From recreational aspect, alpine skiing is one of the most popular winter sports with millions of people participating world-wide [12,13]. Contemporary research literature is mainly related to investigation of physical and physiological characteristics of elite alpine skiers, while research in the field of psychology is scarce, especially in case of recreational alpine skiing [14]. In the present study we examined the influence of fear, worry, self-efficiency and courage on learning alpine skiing in students of kinesiology. It is a popular sport in Croatia, but due to specific environment and weather conditions it is generally considered demanding and challenging [15,16]. Therefore, fear related to new activity and worry about the mentioned exogenous factors can become a psychological barrier that prevents successful learning. Furthermore, in case of inexperienced learners, the mentioned factors can reduce motivation to learn and lead to withdrawal from the activity at an early stage. Finally, fear of injury may cause a behavioral block with refusal to perform specific elements of a ski technique. Therefore, it is important to address different variables such as fear, anxiety, motivation and courage when studying factors influencing sport success and performance [6]. 

We hypothesized that self-efficiency and courage would help students to better acquire new knowledge of alpine skiing. In order to test the psychological factors it is important to have reliable testing possibilities. We therefore used questionnaires with previously quantified metric characteristics in different populations and different countries and adopted them to the Croatian language. For this research, we also specifically developed an instrument Alpine skiing fear inventory [17,18,19]. We tested the metric characteristics of used instruments on young physically capable people, Croatian students of kinesiology with no or only basic previous knowledge of alpine skiing. 

## 2. Materials and Methods

### 2.1. Design and Participants 

The present study included a total of 340 participants (252 alpine ski naive students and 88 students with existing basic knowledge of skiing). They were all third-year students of Faculty of Kinesiology at the University of Zagreb; 106 (31.2%) were females and 234 (68.8%) males (mean age 21.98 ± 1.41 years). We collected data during three consecutive academic years. An absence of any experience in alpine skiing was a definition for an alpine ski naive person. Prior to being enrolled in the study, all participants were informed about the study protocol and aim and gave their written consent to participation. The study was approved by the Ethics Committee of Faculty of Kinesiology. 

### 2.2. Variables 

Alpine ski knowledge was assessed through demonstration of eight previously selected elements of alpine ski technique. Upon completion of structured alpine ski school program, the following elements were graded: traversing left (TL), traversing right (TR), uphill turn to the left (UTL), uphill turn to the right (UTR), snow-plough turn (SPT), basic turn (BT), parallel turn (PT), and short turn (ST). Grades ranged from 1 to 5; where 1 meant an unacceptable level of knowledge and 5 was given for a superb demonstration of an element of the ski technique. Each participant received eight grades and this represented overall knowledge of alpine skiing. Cronbach alpha for skiing performance was 0.91.

Moreover, each eligible participant fulfilled a four-part questionnaire consisting of 65 items. The questionnaire tested the way courage, worry, self-efficiency and fear influence the success in alpine ski learning. Each item was rated on a Likert scale. The questionnaire was not anonymous due to the need for comparison of its results with acquired knowledge of alpine skiing. The questionnaire consisted of the following parts:

(1) Sports Courage Scale (SCS-31) [19]. SCS-31 is a 31-item self-report instrument with 5 subscales titled “Determination, Assertiveness, Mastery, Venturesome and Sacrificial Behavior”. Each item could be answered on a 5-point Likert based scale graded by 1 (I strongly disagree) to 5 (I strongly agree). SCS-31 scale showed adequate validity and reliability in previous research [19].
(2) Penn State Worry Questionnaire (PSWQ) [17]. PSWQ is a 16-item instrument measuring participants’ worry in particular situation. Each item is rated on a four-point scale, ranging from 1 (little) to 4 (strong). The questionnaire has proven validity and reliability in previous research [17]. Cronbach alpha for this research 0.87.
(3) Self-Efficiency in Physical Activity and Alpine Skiing (S-EFPA) [18]. S-EFPA is constructed to assess participants’ self-efficiency. It consists of ten items. Each item is rated on a four-point scale; ranging from 1 (not at all) to 4 (strongly). The questionnaire has a satisfactory validity, but there is a need for more similar studies for its further validation [18]. Cronbach alpha for this research 0.84.
(4) Alpine skiing fear inventory (F & S). F & S is a 9-item instrument used to test fear of alpine skiing. Each item is rated on a 4-point scale ranging from 1 (not at all) to 4 (strongly). Cronbach alpha for this research 0.85.

Since all questionnaires were originally in English, they were translated into Croatian and then translated back to English by a bilingual professional. Moreover, this was the first use of SCS-31 in Croatian population. For this reason, in addition to double translation, we tested the questionnaire on a large sample of participants with similar characteristics (all young, healthy kinesiology students of similar age). We also performed CFA to test the latent structure is in line with original model of SCS-31 (more details available under sections statistical methods and results).

Sport Courage Scale (SCS-31) questionnaire was used for the first time in Croatian population. In order to further explore the factor loadings, exploratory principal axis factoring analysis with oblimin rotation was used. Kaiser–Meyer–Olkin measure of adequacy suggested that the data matrix was suitable for extraction (KMO = 0.926), with Bartlett’s Test of Sphericity Approx. Chi-square = 3900.46; df = 465, *p <* 0.001.

**Figure 1:**
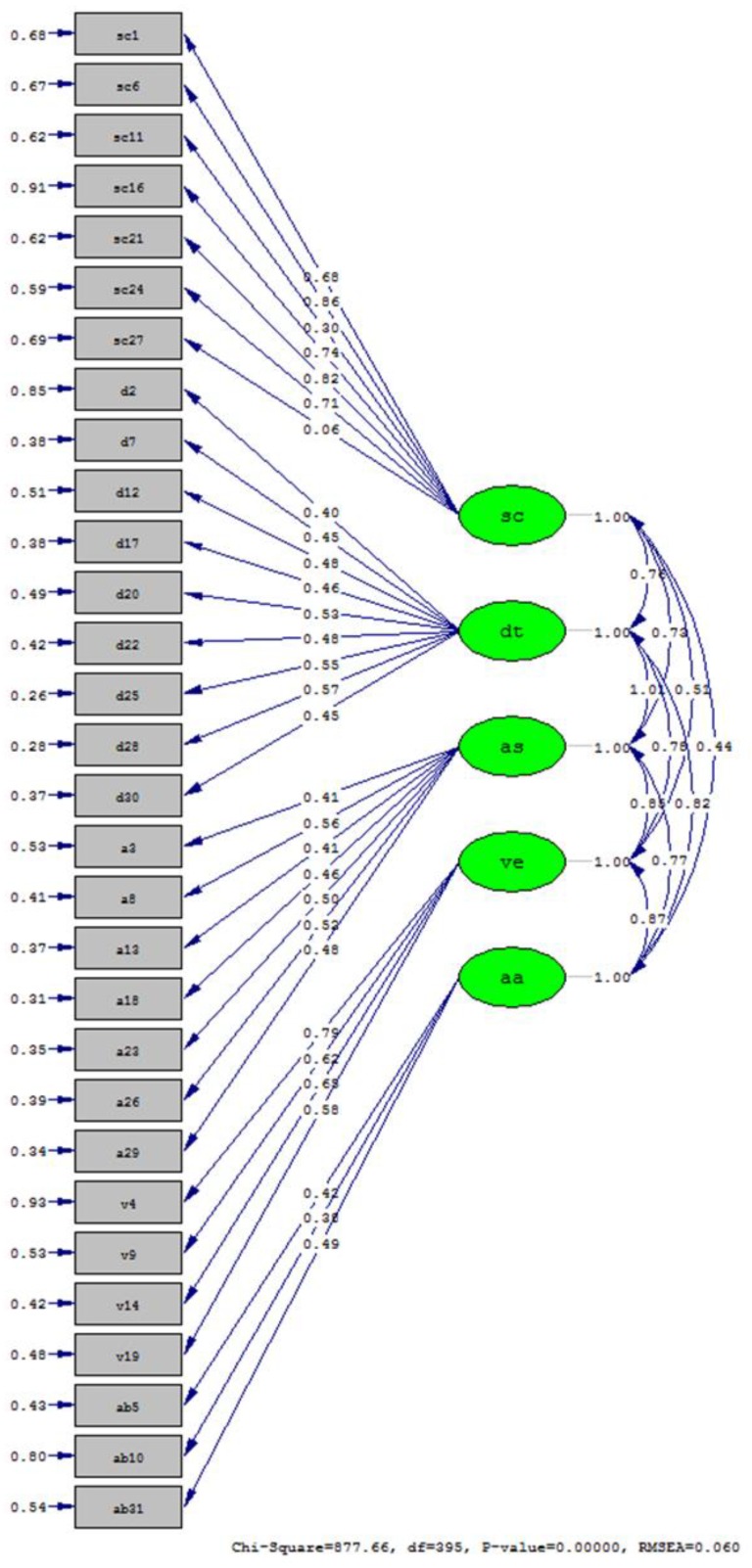
Five factor model of SCS score (sc-mastery; dt-determination; ve-venturesome; as-assertiveness; aa-sacrificial behavior).

Five factors were extracted, explaining a total of 50.4% of variance of SCS; data presented in Figure 1. 

The first factor explains the largest proportion of the variance (31.1%), and items loaded are mostly from the Determination subscale (e.g., “I feel that I have the strength to be successful in difficult conditions”), but also some items form Assertiveness are loading on this factor (“I like to take initiative in the face of difficulties”), indicating less than clear factor structure.

The second factor explains 6.6% of variance, and it consists of items regarding mastery (e.g., “I have limited success because I get frightened”, with reverse coding).

The third factor explains additional 4.7% of variance and it should consist with Assertiveness items (“I have no problems responding to opponent’s sudden attacks”), but it has only 4 items loaded on this factor. 

The fourth factor explained additional 4% and contains majority of Venturesome items (e.g., “I would take any type of risks to become successful”), but additional items load on this factor (like Assertiveness “I assert myself even when facing hazards”, Sacrificial Behavior “I compete even if I have much more to lose than to gain”). 

The fifth factor also additionally explains 4% of variance, but it is a combination of items from Assertiveness “I continue to compete without panicking even when faced with a danger”, Venturesome “Even when facing the possibility of injury, I perform to the best of my ability” and Determination “I perform to the best of my ability no matter how negative the current conditions”. This unclear factor structure indicates the need for further validation of this scale in Croatian athletes. 

Since the sample is limited to athletes involved in skiing, and not various other sports as in the original validation procedure, and due to possible cultural differences, the original model of latent structure is used in this research and the subscales were formed based on the original factors extracted by the authors of the scale. Factors analysis if SPS-31 questionnaire is presented in Table 1.

Confirmatory factor analysis (CFA) conducted on data from the current research to verify the latent structure shows rather good model fit (24.6% of variance explained, CFI (confirmatory factor analysis) = 0.965, RMSA (root mean square error for approximation) = 0.06), justifying the use of originally defined 5 subscales. Both confirmatory and exploratory factor analysis show that one item (“I can take criticism of my principles or believes”) is not saturated on any of the five factors and is excluded from the analysis because it decreases overall subscale internal consistency. Therefore, all except one subscale show very good internal consistency: Cronbach alpha for determination, assertiveness, mastery, venturesome are 0.82, 0.80, 0.77, 0.75, respectively. Only subscale sacrificial behavior shows poor Cronbach alpha 0.47 (only 3 items after item 15 is removed). Sacrificial behavior should be considered and used with caution. Moreover, Cronbach alpha for PSWQ, SEFPA, F & S and skiing performance were as follows 0.87, 0.84, 0.85 and 0.91.

### 2.3. Investigational Protocol 

Research was conducted during three consecutive academic years. Participants were included in a 10-day structured program of alpine skiing school and then filled in the four-part questionnaire. 

Alpine ski school program was identical for all participants; conducted in the same ski center on identical ski slopes. During the ski school, participants were in groups of ten, and learned alpine skiing 6 h each day. Participants rented ski equipment of similar quality, adjusted to specific morphological characteristics. After completing the program of alpine ski school, participants’ knowledge was graded by independent judges. Grades were given for the demonstration of previously selected elements of the alpine ski technique according to the detailed instructions of demonstration layout. Each grade presented the knowledge of demonstrated element of the ski technique and was given by judges with many years of experience in evaluation of ski knowledge. Judges’ objectivity and homogeneity were determined and allowed the use of obtained grades for the assessment of alpine ski knowledge [20]. Each participant received in total eight grades, which was the basis for overall level of acquired alpine ski knowledge. 

### 2.4. Statistical Analysis 

Data was analyzed using SPSS software (IBM SPSS Statistics for Windows, Version 24.0. IBM Corp., Armonk, NY, USA). Descriptive statistics was used to describe the basic features of the sample in this study: proportions for categorical data, and mean+/− standard deviation for normally distributed continuous variables, or median and interquartile range for variables deviating from normal distribution. Spearman’s coefficient of correlation was used within subsamples. Respecting statistical significance of Levene’s homogeneity of variance test, T-test for independent samples was used to test the significance of differences between two independent groups. 

Reliability analysis for scales used in the research was done using Cronbach alpha indicator, while factor structure for SCS-31 was verified using factor analysis: to further explore the factor loadings, exploratory principal axis factoring analysis with oblimin rotation was used. Additionally, confirmatory factor analysis (CFA) was conducted on data to verify the latent structure corresponding to five expected subscales defined by the authors of the SCS-31. Regression analysis was calculated to find independent predictors of skiing performance.

## 3. Results

Prior to data analysis, descriptive statistics were calculated for all measures used, together with indicators of normality of data distribution. Descriptive statistics for used measures is shown in Table 2.

Although data are not normally distributed (all *p <* 0.05), due to large sample size and data measured on an interval scale, parametric statistic T-test for independent samples was used to test the significance of differences between males and females, and between beginners and skiers. When Levene’s test of equality of variances is statistically significant, option “variances not assumed” was used to determine statistically significant differences between given groups. Data are shown in Table 3.

There are statistically significant differences between males and females on several variables. Males score higher on average in Determination, Mastery, Venturesome, Assertiveness and Self-efficiency (all *p <* 0.05). Females have higher average scores in fear inventory (*p <* 0.001). There are statistically significant differences between beginners and skiers on several variables. Experienced skiers score higher on average inn determination, mastery, self-efficiency and skiing performance (all *p <* 0.05), while beginners score higher in fear inventory (*p <* 0.001). Since there are statistically significant differences between males and females and between beginners and skiers, correlations between variables are calculated separately for those subgroups. Data are shown in Table 4. Spearman’s coefficient of correlation was used because it is more appropriate when data is not normally distributed or the relationship between variables is not linear.

In females, Determination and Mastery from SCS-31 have a weak positive correlation with skiing performance (*r =* 0.21, *p <* 0.05 and 0.28, *p <* 0.01, respectively), and so does Self-efficiency (*r =* 0.33, *p <* 0.01). Moderate association between Fear Inventory and skiing (*r =* −0.46, *p <* 0.01) is negative in direction, suggesting females with higher fear have poorer skiing performance. In males, only Self-efficiency is associated with better skiing performance (weak correlation, *r =* 0.39, *p <* 0.01), and as in females, higher fear is related to poorer performance (*r =* 0.33, *p <* 0.01). 

Data in Table 5 show correlations between psychological traits and skiing performance in skiers and ski novices.

For skiers, lower score in PSWQ (*r =* −0.28, *p <* 0.01) and higher score in Self-efficiency (*r =* 0.22, *p <* 0.05) are weakly associated with better skiing performance. For beginners, lower score in Fear (*r =* −0.30, *p <* 0.01) and higher score in self-efficiency (*r =* 0.28, *p <* 0.05) and in mastery (*r =* 0.20, *p <* 0.01) are weakly associated with better skiing performance.

Regression analysis was calculated to see which variables are independent predictors of skiing performance. Since correlation matrix differs for subgroups according to gender and previous skiing experiences, series of regressions are done on each subsample. Method stepwise forward was used, with 0.05 level for entry and 0.10 level of significance for removing the variable, variables entered in blocks: first block subscales from SCS-31, and the second block FEAR, SEFPA and PSWQ (as psychological characteristics). Data are presented in Table 6. 

In both subsamples linear regression analysis shows that entered variables explain significant proportion of variance of the dependent variable, skiing performance (9% of variance in beginners and 12% in skiers, *p <* 0.01 and *p <* 0.05, respectively). For beginners, regression model was analyzed in 4 steps, finally including 3 significant predictors. Independent predictors of skiing performance are: SEFPA (*p* = 0.004), PSWQ (*p* = 0.014), and Fear score (*p* = 0.043) reducing fear and increasing scores on SEFPA and PSWQ increase skiing performance. For skiers, only one predictor has a role in skiing performance: PSWQ score (*p* = 0.004), meaning decreasing worry increases skiing performance.

In gender related subsamples linear regression analysis shows that entered variables explain significant proportion of variance of the dependent variable, skiing performance (16% of variance in males and 18% in females, both *p <* 0.01). For males, regression model was analyzed in 2 steps, finally including 2 predictors, but only one being statistically significant for predicting skiing performance for males: SEFPA (*p <* 0.001)—increasing self-efficiency increases skiing performance. For females, regression model was analyzed in 2 steps, finally including 2 predictors, but only one remaining significant independent predictor of skiing performance for females, and that is Fear score (*p <* 0.001) decreasing scores in fear increase skiing performance.

## 4. Discussion

Alpine skiing, although enjoyed by millions of people worldwide, is perceived as a “high-risk” sport [21], demanding for both learning and teaching [22]. Among different factors that can affect skiing, personality traits are highly important. Up to date, they were more often investigated in competitive level skiers, and recently more widely with respect to skiing-related injuries. Research by Johansson and co-authors (2015) suggests that alpine skiers with previous injuries differ from those not injured in the trait stress susceptibility, which is higher among the latter and thus perceived as a protective trait [16]. It seems that psychological traits defined in male skiers correlate with more risky behavior and therefore higher burden of injury [23]. This broadened the investigation of risk-taking behaviors and characteristics of participants in winter sports to set educational campaigns and reduce accident rates [24]. We believe it is an important area to investigate also from the perspective of future ski instructors, who will set example for their alpine ski school participants on how to learn alpine skiing more efficiently and safely. 

Our previous research suggests traits such as self-efficiency and confidence can help in learning alpine skiing, especially more complex elements of the ski technique [15]. Others have shown how anxiety diminishes effectiveness of alpine ski instruction [25]. 

In this research we investigated sport courage defined through determination, mastery, assertiveness, venturesome, altruistic behavior and self-efficiency as positive traits known to influence attitude towards sport and sport success on one hand [7] and on the other hand fear and worry as traits with potential to limit sports performance [6,26]. The study included only kinesiology students, with either no previous knowledge or only basic knowledge in alpine skiing. From the aspect of alpine skiing, to be daring, venturesome, and willing to participate in difficult situations could be an important trait to differ skiers with better performance. Similarly, from the aspect of alpine skiing, determination is an important trait because it is a quality that makes one continue in trying, even though it might be difficult, especially during initial phases of ski learning. Mastery is power, control and in skiing it might be control over speed for example, which is important for ski beginners as it can lead to reduction of fear. Assertiveness is defined as confidence, self-assurance which from the skiing perspective relates to better success in learning process and is also important for advancement. Self-efficiency is closely related to capability of succeeding in a specific task and is important for ski beginners as well as competitors. Fear is an unpleasant emotion caused by being aware of danger, and as skiing is by its nature a sport that tests the boundaries of thrill and fear, it can be under negative influence of fear. When addressing fear in terms of alpine skiing, it can encompass many different things and situations; from fear of speed, fear of injury to fear of crowded ski-slopes or ski-lifts, fear of snow conditions or weather to fear of looking foolish while trying to learn alpine skiing. Worry means being anxious or troubled about actual or potential problems, which again in skiing can be multifactorial. 

Understanding and overcoming undesirable traits or in case of positive traits their accentuation may help in selection of adequate didactic methods and result in more efficient learning [6]. In our research we found differences in psychological traits between alpine ski naive athletes and those with previous basic knowledge of alpine skiing. Already experienced skiers scored higher in determination, mastery, self-efficiency. At the same time beginners scored higher in fear inventory. The mentioned facts suggest how positive previous experience influences attitude towards new activity and can help in better learning. Research by Kintschera and coworkers showed that ski beginners worry about falling and keeping balance during initial phases of alpine ski learning, which is influenced by fear and lower self-efficiency [27] and may limit the speed and results of ski learning. On the other hand, experienced skiers with positive psychological traits achieve better results during evaluation of skiing performance. 

According to published data, one can also expect differences between males and females in psychological traits linked to sport success [18,27]. Literature suggests males to be more self-efficient and confident and ready to take more risks, all of which transfers to success and better sport performance. Gender-related differences are especially notable in traits self-efficiency and fear, which according to Giulianou and co-workers has its roots in cultural stereotypes where males receive more attention and support from family, coaches and trainers than their female counterparts, predisposing them to better sport results and taking part in high-risk sports [28]. 

Although our research included young athletes of similar interest in sport and one would not expect such pronounced differences in psychological traits, we did notice statistically significant differences between male and female athletes in sport courage related traits. The mentioned facts were all more pronounced in males, and represent potentially more positive attitude of males towards sport and skiing. Similarly, Konter describes male sport participants to be more determined, assertive and venturesome than female counterparts [29]. Moreover, our female participants expressed more fear than males, which is also noted in other sports [18]. Fear is an important trait which might limit learning a new motor activity [30]. According to our results, female participants with more expressed fear had poorer skiing performance than those who were more determined and had greater scores in mastery, and self-efficiency. Interestingly, in males, only self-efficiency was associated with better skiing performance, but as in females, higher fear was related to poorer performance. Similar, detrimental effects of fear on sport success were also described in other sports [31,32]. 

Regardless of gender, participants with previous ski experience who scored lower in worry and higher in self-efficiency had better skiing performance. At the same time, ski beginners with lower score in fear and higher score in self-efficiency and mastery were better at alpine ski learning. Independent predictors of skiing performance for ski beginners are self-efficiency and worry which when increased relate to better performance and at the same time fear which when reduced also helps skiing performance. For skiers, only worry had an influence on skiing performance, meaning decreasing worry increases skiing performance. For males, significant predictor of skiing performance is self-efficiency which when increased leads to better skiing performance. For females, significant independent predictor of skiing performance was fear, suggesting that decreasing fear might improve skiing performance. Similar results showing positive effects of self-efficiency and confidence on reduction of fear of injury are shown by different studies and can help in learning a new motor activity and motivation [33]. Finally, it is important to mention study limitations, primarily related to a sample consisting of only young motorically capable people. In the future research and for the research to have a more practical impact on a broader population it would be necessary to include participants of different age and motor abilities.

In conclusion, psychological factors need to be considered during learning phases of alpine skiing. To improve ski learning, teachers and ski instructors would need to help ski beginners increase self-efficiency and reduce fear. Additionally, possible gender differences in psychological traits need to be considered. When approaching male ski beginners self-efficiency is associated with better performance, while in female ski beginners it is important to reduce fear to achieve better results in basics alpine ski school program, as there is a negative association between the two. Mentioned approach could not only improve but also speed up the learning process. 

## Figures and Tables

**Table 1:** Factor analysis of SPS-31 questionnaire.

Questionnaire	Factor				
	1	2	3	4	5
scs8	0.720				
scs29	0.619				
scs17	0.617				
scs18	0.552				
scs30	0.541				
scs25	0.528	−0.223			
scs28	0.506	−0.239			
scs7	0.374				
scs15					
scs16		0.694			
scs6		0.664			
scs21		0.645			
scs1		0.523			
scs24	−0.319	0.474			
scs11		0.399			
scs22		−0.343	0.235	−0.263	
scs13		−0.254	0.452	−0.212	
scs2			0.381		
scs3		−0.208	0.283	−0.234	
scs27			0.266		
scs4				−0.677	
scs9				−0.539	0.231
scs14				−0.502	0.259
scs10				−0.493	
scs23	0.298			−0.451	
scs31				−0.398	
scs5			0.306	−0.382	
scs19				−0.267	0.555
scs26	0.370				0.385
scs12	0.219	−0.201			0.365
scs20				−0.230	0.351

**Table 2:** Mean values and standard deviations, median and interquartile range, and normality of distribution indicator are shown for each scale and subscale.

Questionnaire	M	SD	Min	Max	Median	Percentile 25	Percentile 75	P (Kolmogorov-Smirnov Z)
SCS-31								
Determination	34.62	4.76	19	45	35	32	38	<0.001
Mastery	22.77	4.78	10	34	23	19	26	<0.001
Venturesome	13.21	3.06	4	20	13	11	15	<0.001
Assertiveness	26.12	3.71	14	35	26	24	28	<0.001
Sacrificial Behavior	11.50	1.84	5	15	12	10	13	<0.001
PSWQ	45.24	9.69	22	72	44	39	51	<0.001
SEFPA	38.64	5.89	22	50	39	35	43	<0.001
F & S	14.55	5.40	8	39	13	11	16	<0.001
Skiing Performance	29.77	5.17	18	40	30	26	33.50	<0.05

SCS-31-Sports courage scale; PSWQ: Penn State Worry Questionnaire; SEFPA: Self-efficacy in physical activity and alpine skiing; F & S: Alpine skiing fear inventory; Skiing performance: knowledge of demonstrated element of ski technique.

**Table 3:** Differences in skiing performance and psychological variables regarding gender and previous skiing experience.

**Gender**		***n***	**Mean**	**Std. Deviation**	***p***
Determination	male	234	35.20	4.36	0.002 *
	female	105	33.31	5.34	
Mastery	male	234	23.20	4.52	0.019 *
	female	106	21.81	5.22	
Venturesome	male	234	13.60	2.91	<0.001 *
	female	105	12.34	3.22	
Assertiveness	male	234	26.53	3.42	0.005 *
	female	106	25.22	4.15	
Sacrificial Behavior	male	234	11.63	1.76	0.054
	female	106	11.22	1.99	
PSWQ	male	230	44.92	9.57	0.378
	female	104	45.93	9.94	
SEFPA	male	230	39.57	5.53	<0.001 *
	female	106	36.60	6.15	
F & S	male	232	13.40	4.27	<0.001 *
	female	106	17.08	6.64	
Skiing Performance	male	238	29.70	5.03	0.690
	female	106	29.94	5.49	
**Skiing Previously**		***n***	**Mean**	**Std. Deviation**	***p***
Determination	beginners	249	34.31	5.06	0.017 *
	skiers	88	35.52	3.66	
Mastery	beginners	250	22.41	5.02	0.012 *
	skiers	88	23.75	3.97	
Venturesome	beginners	250	13.34	3.15	0.217
	skiers	87	12.87	2.75	
Assertiveness	beginners	250	25.98	3.84	0.192
	skiers	88	26.58	3.28	
Sacrificial Behavior	beginners	250	11.55	1.90	0.511
	skiers	88	11.40	1.66	
PSWQ	beginners	248	45.33	9.96	0.791
	skiers	85	45.01	8.95	
SEFPA	beginners	246	37.68	5.76	<0.001 *
	skiers	88	41.23	5.37	
F & S	beginners	248	15.36	5.82	<0.001 *
	skiers	88	12.33	3.11	
Skiing Performance	beginners	250	28.35	4.62	<0.001 *
	skiers	89	34.00	4.22	

PSWQ—Penn State Worry Questionnaire; SEFPA—Self-efficacy in physical activity and alpine skiing; F & S—Alpine skiing fear inventory; Skiing performance—knowledge of demonstrated element of ski technique. * *p* < 0.05.

**Table 4:** Spearman’s coefficient of correlation between variables for female sample (*n* = 105) and for male sample (*n* = 235).

**Females (*n* = 105)**	**Mastery**	**Venturesome**	**Assertiveness**	**Sacrificial Behavior**	**PSWQ**	**SEFPA**	**Fear**	**Skiing Performance**
Determination	0.69 **	0.57 **	0.80 **	0.43 **	−0.29 **	0.56 **	−0.48 **	0.21 *
Mastery		0.44 **	0.66 **	0.29 **	−0.49 **	0.55 **	−0.46 **	0.28 **
Venturesome			0.66 **	0.41 **	−0.16	0.28 **	−0.35 **	0.07
Assertiveness				0.41 **	−0.38 **	0.47 **	−0.44 **	0.13
Sacrificial Behavior					−0.05	0.18	−0.18	−0.07
PSWQ						−0.34 **	0.24 *	−0.17
SEFPA							−0.46 **	0.33 **
F & S								−0.46 **
**Males (*n* = 235)**	**Mastery**	**Venturesome**	**Assertiveness**	**Sacrificial Behavior**	**PSWQ**	**SEFPA**	**Fear**	**Skiing Performance**
Determination	0.45 **	0.50 **	0.80 **	0.52 **	−0.19 **	0.44 **	−0.36 **	0.12
Mastery		0.27 **	0.49 **	0.22 **	−0.46 **	0.34 **	−0.44 **	0.14 *
Venturesome			0.49 **	0.52 **	−0.07	0.18 **	−0.25 **	−0.03
Assertiveness				0.41 **	−0.21 **	0.36 **	−0.31 **	0.09
Sacrificial Behavior					−0.074	0.23 **	−0.23 **	−0.06
PSWQ						−0.27 **	0.33 **	−0.02
SEFPA							−0.47 **	0.39 **
F & S								−0.33 **

PSWQ—Penn State Worry Questionnaire; SEFPA—Self-efficacy in physical activity and alpine skiing; F & S-Alpine skiing fear inventory; Skiing performance- knowledge of demonstrated element of ski technique. ** *p <* 0.01, * *p <* 0.05.

**Table 5:** Spearman’s coefficient of correlation between variables for skiers (*n* = 88) and ski beginners (*n* = 249).

**Skiers (*n* = 88)**	**Mastery**	**Venturesome**	**Assertiveness**	**Sacrificial Behavior**	**PSWQ**	**SEFPA**	**Fear**	**Skiing Performance**
Determination	0.42 **	0.47 **	0.76 **	0.34 **	−0.17	0.47 **	−0.21 *	0.15
Mastery		0.24 *	0.42 **	0.15	−0.35 **	0.52 **	−0.17	0.05
Venturesome			0.56 **	0.42 **	−0.08	0.26 *	−0.19	0.05
Assertiveness				0.38 **	−0.32 **	0.44 **	−0.19	0.09
Sacrificial Behavior					−0.08	0.22 *	−0.12	0
PSWQ						−0.32 **	0.28 *	−0.28 **
SEFPA							−0.32 **	0.22 *
F & S								−0.13
**Ski Beginners (*n* = 249)**	**Mastery**	**Venturesome**	**Assertiveness**	**Sacrificial Behavior**	**PSWQ**	**SEFPA**	**Fear**	**Skiing Performance**
Determination	0.58 **	0.59 **	0.81 **	0.55 **	−0.24 **	0.49 **	−0.46 **	0.103
Mastery		0.40 **	0.59 **	0.28 **	−0.51 **	0.40 **	−0.51 **	0.20 **
Venturesome			0.57 **	0.52 **	−0.12	0.27 **	−0.38 **	0.03
Assertiveness				0.44 **	−0.26 **	0.41 **	−0.43 **	0.07
Sacrificial Behavior					−0.07	0.26 **	−0.27 **	−0.07
PSWQ						−0.31 **	0.30 **	−0.02
SEFPA							−0.48 **	0.28 **
F & S								−0.30 **

PSWQ: Penn State Worry Questionnaire; SEFPA: Self-efficacy in physical activity and alpine skiing; F & S: Alpine skiing fear inventory; Skiing performance: knowledge of demonstrated element of ski technique. ** *p <* 0.01, * *p <* 0.05.

**Table 6:** Regression coefficients for predictors of skiing performance, regarding previous skiing experience (beginners and skiers) and gender.

**Questionnaire**	**Beginners (*n* = 244)**			**Skiers (*n* = 84)**		
	**Standardized Coefficients (Beta)**	**t**	***p***	**Standardized Coefficients (Beta)**	**t**	***p***
Mastery	0.120	1.518	0.130			
SEFPA	0.213	2.933	0.004 *			
PSWQ	0.160	2.271	0.024 *	−0.313	−2.982	0.004 *
F & S	−0.156	−2.035	0.043 *			
	R^2^ = 0.13, R^2^Adj = 0.12, F = 8.996, *p <* 0.001			R^2^ = 0.10, R^2^Adj = 0.09, F = 8.893, *p* = 0.004		
	**Males (*n* = 227)**			**Females (*n* = 102)**		
	**Standardized Coefficients (Beta)**	**t**	***p***	**Standardized Coefficients (Beta)**	**t**	***p***
Mastery	0.021	0.319	0.750	0.079	0.762	0.448
SEFPA	0.402	6.141	<0.001 *			
F & S				−0.396	−3.804	<0.001 *
	R^2^ = 0.17, R^2^Adj = 0.16, F = 22.663, *p <* 0.001			R^2^ = 0.20, R^2^Adj = 0.18, F = 11.986, *p <* 0.001		

* *p* < 0.05.
